# Crypto-Crime Tumblers: Criminogenic Attributes of Cryptocurrency Mixers in Enabling Financial Crime and Money Laundering

**DOI:** 10.1177/10439862261487891

**Published:** 2026-09-28

**Authors:** Wesley Kwan, Christian Leuprecht

**Affiliations:** 1 Queen’s University, Kingston, Ontario, Canada; 2 Royal Military College of Canada, Kingston, Ontario, Canada

**Keywords:** cryptocurrency, money laundering, anti-money laundering, mixer, tumbler

## Abstract

Cryptocurrency mixers have played a growing role in facilitating money laundering. This article examines 32 mixer-related court cases to identify criminogenic patterns in offender roles, laundering practices, and enforcement outcomes. Results indicate that mixer-enabled cryptocurrency money laundering is concentrated among a small number of services, with five mixers linked to over $6 billion in illicit funds. Non-custodial mixers processed the majority of total cryptocurrency volume, while centralized mixers transferred most funds tied directly to predicate criminal offenses. Laundering schemes were dominated by Bitcoin and Ether, with altcoins playing ancillary roles. The identified core actor types—overseers, personal users, and enablers—highlight the networked and multi-layered structure of mixer-based laundering. Decentralized services, regulatory arbitrage, and rapid mixer substitution following takedowns contribute to mixer resilience. Reactive enforcement strategies experience limitations. Proactive, internationally coordinated responses to mixer-enabled financial crime are needed.

## Introduction

The rapid diffusion of cryptocurrency is reshaping the landscape of financial crime. Criminal justice systems are designed around centralized, institution-based models of regulation and enforcement. Among the most consequential innovations is the rise of cryptocurrency mixers: services that intentionally obfuscate transaction histories by pooling and redistributing digital assets. Although mixers can serve lawful privacy-enhancing purposes, they have become implicated disproportionately in money laundering, ransomware, darknet market activity, and other forms of cyber-enabled crime. Criminal justice responses have largely focused on sanctions, prosecutions, and regulatory classification. Yet, these interventions are reactive and uneven, particularly when applied to decentralized or non-custodial mixing services. Technical detection methods or normative debates over privacy and regulation dominate current scholarly debate, yet provide few insights into how mixers actually function in real-world laundering schemes and criminal networks. This article addresses that gap by examining documented court cases involving cryptocurrency mixers to identify recurring patterns in offender roles, laundering practices, and enforcement outcomes. By providing an empirical analysis of legal cases, the article situates cryptocurrency mixers in broader debates on financial crime, regulatory failure, and the adaptive dynamics between offenders and contemporary systems of criminal justice.

A significant portion of cryptocurrency transactions are tied to crime. In 2024, an estimated $51.3 billion in cryptocurrency was associated with illicit actors (Chainalysis, 2025). Unlike fiat currency, its decentralized and pseudonymous attributes enable criminals to engage in cryptocurrency money laundering (CML). Similar to laundering fiat currency, CML involves converting “dirty” cryptocurrency derived from predicate offenses into “clean” cryptocurrency that can be integrated into the regular financial system (Hamilton & Leuprecht, 2024; Leuprecht et al., 2023). The rise in cryptocurrency mixers (also known as “tumblers”) has supercharged CML. For example, the popular Bitcoin Fog mixer was found to have laundered hundreds of millions of dollars in proceeds from narcotics trading, trafficking in child sexual abuse material, identity theft, and other illicit activities (U.S. Department of Justice, 2024). Cryptocurrency mixers facilitate the laundering process by “mixing” funds of many users and distributing them to new addresses, thereby obscuring the cryptocurrency’s original source. This process makes it difficult for investigators to trace illicit funds to their ultimate destination. While enhanced privacy has legitimate uses (e.g., protecting personal financial privacy), it is also disproportionately associated with criminal activity: in 2022, approximately 23% of funds sent to mixers came from illicit addresses (Chainalysis, 2022a), while only 0.42% of overall cryptocurrency transaction volume was tied to illicit activity (Chainalysis, 2024a). Designed for centralized, fiat-based financial systems, anti-money laundering (AML) frameworks are struggling to adapt to the decentralized, pseudonymous nature of cryptocurrency. Prominent policymakers and regulators, such as the Financial Action Task Force (FATF), the Financial Crimes Enforcement Network (FinCEN), and the Office of Foreign Assets Control (OFAC), have begun to sanction and regulate mixers. But enforcement remains reactive and inconsistent across jurisdictions, and mixers have proven adept at exploiting regulatory arbitrage.

This article examines the role of cryptocurrency mixers in CML schemes and responses by regulators and law enforcement. Specifically, what insights can real-world mixer cases offer into containing mixer-enabled CML? Initially, this article provides context to inform our understanding of cryptocurrency and mixers. Second, it reviews the literature on mixers and CML. Then, the article sketches a more detailed overview of the way mixers have been regulated and treated by law enforcement and identifies gaps in knowledge. Following the methodology, the article draws on a medium-*n* body of documented court cases to identify common patterns and trends in how mixers facilitate CML and to evaluate the efficacy of current regulatory and enforcement measures. The analysis also highlights three case studies that are representative of the wide scope of CML mixer cases. The article concludes with broader implications and areas of focus.

## Background

Cryptocurrency is a form of digital currency that can be used for purchases, trading, and investments, with transactions recorded on a virtual public ledger referred to as a “blockchain” (Jenkins et al., 2023). By way of exchanges, customers can trade cryptocurrencies for other cryptocurrencies or assets such as fiat money. The cryptocurrency environment is heterogeneous: Bitcoin and Ether are among the most popular cryptocurrencies. While both operate on public, permissionless blockchains where transaction data are openly visible, other digital assets may operate on permissioned or privately governed networks (Tan et al., 2022). Some stablecoins, such as Tether, are centrally issued and retain administrative control over token balances. Accordingly, “cryptocurrency” does not uniformly imply decentralization. This article focuses primarily on public blockchain systems, where mixers feature prominently in CML cases.

Blockchains such as Bitcoin are pseudonymous. Users are identified only by their cryptographic address, which functions as their pseudonym. Though difficult, law enforcement and investigators can analyze transaction patterns and correlate blockchain data to external information in an attempt to link addresses to real-world identities. However, mixers hinder these investigative techniques. Mixers obfuscate the transaction trail by combining cryptocurrency from multiple users and redistributing it to a new address such that a user’s funds are decoupled from their original deposit (Tippe & Deckers, 2025). Mixers typically charge a fee, often a percentage of the mixed amount, for their services.

Approaches to mixing cryptocurrency can be classified as “custodial” or “centralized” and “non-custodial” (Rodwald, 2025). With centralized custodial mixers, a third party receives the cryptocurrency assets, mixes them with other users’ assets, and redistributes them. Given the reliance on centralized operators, if the service is compromised, users’ anonymity is breached. Decentralized mixers operate through a non-custodial, open-source protocol, whereby users submit their assets to the protocol and receive a cryptographic key in return, which can later be used to designate a destination address. Meanwhile, assets are mixed. To withdraw funds from the mixer, users present the cryptographic key, which prompts the protocol to transfer the funds to a new address (Finger & Ramachandran, 2023). No third party takes custody of the funds during the process, which makes such services difficult to regulate. Some cryptocurrency wallets implement CoinJoin, a decentralized, non-custodial technique that mixes users’ funds in a single transaction (Rodwald, 2025). This phenomenon is an example of a centralized mixing service using a decentralized protocol, in which users retain control of funds but still rely on a central coordinator (Wu et al., 2021). Such services with both centralized and decentralized aspects are best described as coordinator-based, non-custodial mixers.

## Overview of Literature

### Cryptocurrency Mixers and CML

Conventional CML follows the traditional money laundering process of placement, layering, and integration (Holt et al., 2023; Liu et al., 2021). Placement aims to cut off the connection between the precursor crime and the derived funds by introducing the funds into the financial system. This involves breaking down large funds into smaller ones. Layering entails distributing funds across different accounts and institutions to obfuscate the original source, which is characterized by techniques such as chain hopping and peel chains. Mixers play a key role in CML at this stage. Finally, integration involves incorporating funds into the legitimate economy, thereby “legalizing” illicit funds for use in transactions. Virtual Asset Service Providers (VASPs) typically serve as an off-ramp at that stage of CML.

A goal-oriented framework conceptualizes roles in CML mixing (Liu et al., 2021). Agents operate in a structured hierarchy to move illicit funds across borders while avoiding detection. At the core of the CML process, Organizers (commonly known as Controllers) allocate resources and organize laundering pathways using communicators and soldiers as intermediaries; they are most prevalent at the integration stage. Communicators (commonly known as Transmitters) reside at the middle level: they transmit laundering information by communicating with customers and transferring funds across accounts. They are prevalent at the layering stage and work together with organizers during integration. Soldiers (commonly known as Collectors) are employed by organizers to facilitate the CML process by splitting and obfuscating funds in the placement and layering stages. Tiwari et al. (2023), however, deem the traditional placement-layering-integration framework outdated and advocate a more holistic model. Their Actors involved, Predicate crime, the Purpose of laundering, and Technological innovations (APPT) framework is characterized by (a) actors’ knowledge and skills insofar as they help determine their role in the scheme, (b) forms of predicate crime, (c) laundering aims, which are influenced by the ideology of actors, and (d) the use of evolving technologies. CML cases illustrate all four dimensions.

Different mixers vary in how effectively they obfuscate the source of funds. An early study of three mixers by Möser et al. (2013) traced the flow of transactions from origin to destination for BitLaundry, while Bitcoin Fog and Blockchain.info were successful in anonymizing transactions. Other studies have demonstrated security and privacy flaws in major mixers, including AlphaBay and Helix. Users are left with a false sense of security, while law enforcement leverages these deficiencies to trace CML (de Balthasar & Hernandez-Castro, 2017; Pakki et al., 2021). Rodwald (2025) analyzed six mixing services and found it impossible to establish a correlation between an input and output address. Three mixers (i.e., YoMix, Sinbad.io, and Coinomize) left no evidence suggesting that a mixer had even been used in the transactions. Whereas blockchain analysis company Chainalysis (2022a) has expressed optimism in developing the capabilities to demix transactions and thus render mixers obsolete, evidence suggests that mixers remain effective at obfuscating and anonymizing cryptocurrency transactions, notwithstanding gaps in security (See, 2023).

### Regulations and Enforcement

Using cryptocurrency mixers is not inherently unethical. Legitimate users may seek privacy for sensitive transactions or protection from authoritarian regimes (Tippe & Deckers, 2025). However, the very features that protect a user’s privacy are also inherently criminogenic: from 2020 to 2021, across 108 reports under the Bank Secrecy Act, FinCEN (2024) found that mixers were used to help conceal transactions in connection with online child sexual exploitation or human trafficking. As a result, regulators face a conundrum, as methods that deanonymize or ban mixers would also erode financial privacy rights for lawful users. Indeed, mixers would lose their value should they permit regulators to deanonymize CML transactions, since it would then be possible to deanonymize legitimate transactions. To balance privacy and legality, See (2023) has proposed using zero-knowledge proofs that allow authorities to extract transaction chains of only known tainted mixer transactions, which avoids having to divulge privacy information of legitimate users. Nevertheless, the policy trajectory now deems mixers as CML enablers (U.S. Department of Justice, 2020).

In most jurisdictions, cryptocurrency mixers are not explicitly illegal, but their operations are often subject to financial regulations. In the United States, centralized or custodial mixers are deemed money services businesses under the *Bank Secrecy Act* and must (a) register with FinCEN, (b) maintain an AML and know-your-customer (KYC) compliance program, and (c) meet reporting and record-keeping requirements, such as retaining customers’ Internet Protocol (IP) addresses (Chainalysis, 2022b; U.S. Department of Justice, 2020; U.S. Secret Service, 2025). Many custodial mixers have not followed U.S. regulations, as doing so would likely cause them to lose customers. Instead, mixers often cease operations in the United States while still accepting U.S. customers, which leaves them subject to law enforcement action.

In 2020, Larry Dean Harmon received the first-ever FinCEN penalty against a mixer for operating Helix. He was fined $60 million for violating *Bank Secrecy Act* regulations and enabling over $300 million in CML (FinCEN, 2020). He had been criminally charged in 2019 and later pleaded guilty. In 2022, Blender.io became the first cryptocurrency mixer sanctioned by the U.S. Treasury’s OFAC. It was used by Lazarus Group, a North Korean state-sponsored cyber hacking group that laundered over $20.5 million from the Axie Infinity Ronin bridge hack (OFAC, 2022). That same year, Tornado Cash became the first sanctioned non-custodial or decentralized mixer, which marks an enforcement shift from centralized entities and persons (Brownworth et al., 2024). Enforcement strategy against CML has centered around taking down mixers and prosecuting their operators and users (see Appendix).

This enforcement strategy has met with some success. Following sanctions on Tornado Cash, their transaction volumes dropped by around 72% (Brownworth et al., 2024), and after their web-based interface was later taken down, transaction volumes dropped by 90% (Chainalysis, 2025). Owing to the implementation of more robust AML/KYC policies and the takedown of non-compliant services, preferred methods for laundering ransomware payments have been shifting away from mixers and centralized exchanges (Chainalysis, 2024a). Between 2022 and 2023, overall illegal funds sent to mixers declined from $1.01 billion to $504.3 million (Chainalysis, 2024a).

### Enforcement Challenges and Gaps

Challenges and gaps in enforcing AML regulations against mixers persist. Notably, decentralized mixers do not face the same vulnerability to seizure and investigation as centralized mixers, as they do not have central operators or servers from which to retrieve logs and their infrastructure is difficult to shut down (Chainalysis, 2025; See, 2023). Cryptocurrency inflow to Tornado Cash in 2024 surged by 108% year-over-year, despite its web-based interface being taken down (Brownworth et al., 2024; Chainalysis, 2025). Sanctions against Tornado Cash were removed in March 2025 over legal challenges to the government’s latitude to sanction decentralized protocols (U.S. Department of the Treasury, 2025).

In addition, proposed recommendations for information standardization through collaboration between law enforcement and mixing services (Carletti et al., 2024) or harsher punishments for non-compliance (Leuprecht et al., 2023) run up against operators who are unlikely to yield to authorities. Mixer operators tend to be motivated by their philosophical outlook rather than monetary or pragmatic concerns (Brownworth et al., 2024). Mixers are a manifestation of the emancipatory cryptoanarchist foundation of cryptocurrencies: a radically free, private, and self-regulating transaction system (Chohan, 2017a, 2017b). Accordingly, as soon as one illicit mixer is shut down, an alternative mixer appears, which gives rise to a diffusion effect. After Tornado Cash was sanctioned, Sinbad.io became the Lazarus Group’s go-to mixer until its takedown, resulting in YoMix now serving as its replacement for laundering illicit funds (Chainalysis, 2024a). Lazarus Group has also shifted to using Kruw.io to launder proceeds from the $1.5 billion Bybit hack after Wasabi Wallet stopped offering CoinJoin services (U.S. Secret Service, 2025). Consequently, reliance on takedowns alone is insufficient. Some cybercrime prevention research has explored disruption strategies aimed at prospective offenders, including warning banners and other interventions that seek to increase perceived risk before illicit transactions occur (Moneva et al., 2023). Such approaches may complement traditional enforcement by targeting demand for mixing services in addition to their supply.

Moreover, the global nature of cryptocurrency and mixers raises cross-jurisdictional regulatory heterogeneity. International compliance with the FATF AML recommendations for VASPs varies. As of June 2025, 27% of countries have not implemented a requirement for VASPs to obtain and disclose details related to the sender and recipient for cryptocurrency transactions, 24% of countries have not conducted a risk assessment for VASPs, and 18% of countries have not identified regulations for the cryptocurrency sector (FATF, 2025). As a result, jurisdictions that are slow or weak to regulate become a safe harbor for illicit mixers and criminal networks. Still, the mere presence of AML regulations is insufficient without comprehensive enforcement and adherence. Despite being required to implement AML/KYC protocols, centralized exchanges are still essential in CML mixing operations, particularly at the beginning and end of transaction chains (Zola et al., 2025). These exchanges continue to be a prominent vehicle for integrating ransomware funds into fiat currency (Chainalysis, 2025), which raises doubts about the adequacy of their AML and KYC policies. Since mixers have proven difficult to regulate, Holt et al. (2023) posit the level of the exchange as the only viable prevention mechanism, and van Wegberg et al. (2018) propose that authorities use the opportunity to gather user evidence at exchanges to inform their AML strategy. While Tippe and Deckers (2025) agree on the importance of regulating cryptocurrency exchanges, they also emphasize the need for a multifaceted enforcement approach that combines blockchain analysis and conventional investigative methods to counter CML. Burgess et al. (2024) stress that any investigative protocol should involve cooperation with third-party companies.

### Research Contributions

There have been several experimental analyses of mixing services with an aim to develop algorithms to identify mixing patterns and cryptocurrency flows through mixer transactions (de Balthasar & Hernandez-Castro, 2017; Liu et al., 2021; Möser et al., 2013; Shojaeinasab et al., 2023; Wu et al., 2021). Wu et al. (2022) observed that mixing services tended to (a) receive input transactions before disbursing cryptocurrency, (b) rarely reuse the same address, (c) disburse tainted cryptocurrency to other addresses, and (d) have input transaction amounts equal to output transaction amounts. However, data in these studies are sparse on how mixers are used in the real world beyond experimental transactions, and results are typically specific to analyzed mixing services. These studies focus on the technical mixing mechanism and do not inform the broader context of CML, including preferences and actions of cybercriminals.

Another research approach to address CML mixing is to ascertain patterns involving real-world CML cases. Qualitative analyses of select CML mixer cases draw on small samples and lack quantitative data to identify broader trends in how mixers are used (Carletti et al., 2024; Gable, 2021; Tippe & Deckers, 2025). As a result, there is a dearth of quantitative systemic research on CML cases involving mixers. This article aims to fill this gap by analyzing a medium-*n* dataset of court cases to identify common trends in CML involving mixers. Rather than relying on technical experimentation or theoretical models of cryptocurrency mixing, the article provides an empirical account of how mixers are deployed in real-world criminal enterprises and enforcement responses in practice. Whereas traditional money laundering scholarship is premised on centralized institutions and compliance (Levi & Reuter, 2006), CML operates on fundamentally different principles: a small subset of mixers dominates laundering volumes, decentralized protocols are resistant to takedown, and illicit transactions are increasingly facilitated by smaller cryptocurrencies. By documenting these patterns, this article contributes a novel empirical perspective to a largely theoretical and experimental body of CML research and distinguishes it from conventional money laundering approaches. The resulting data can better inform AML practices and future research.

## Methods

This article is based on an original dataset of 32 documented CML court cases in which cryptocurrency mixers played a role (see Appendix). Cases were collected from public court records from the U.S. Department of Justice website and other legal databases, including Justia, CourtListener, and CanLII. Press releases, news articles, academic papers, and other media reports were used to supplement case information. Searches used a combination of key terms including “mixer,” “tumbler,” “crypto,” and “laundering,” as well as their cognates. Inclusion criteria for cases included the use of cryptocurrency, a charge or clear component of CML, and the involvement of mixers. Both civil and criminal court cases were included to maximize scope.

Variables our instrument coded for include case jurisdiction, date, case outcome, cryptocurrency type, cryptocurrency amount transferred in total and from illegal sources, mixers involved in CML, mixer type (custodial vs. non-custodial), mixer country, start and end dates of mixer activity, and the agents involved in the CML scheme. These variables serve to uncover general patterns or relationships among cases to improve our understanding of CML with mixers. This design is representative of a medium-*n* comparative case-based analysis and is derived from similar studies conducted by Leuprecht et al. (2015) and Leuprecht et al. (2023). The data were analyzed using frequency tables.

Given the decentralized nature of CML cases, actors within networks are often unidentified or serve many different roles, which limits the opportunity for mathematical clustering or network analysis (Leuprecht et al., 2023). The article is also limited in that some case information was omitted due to redactions and insufficient detail in court documents. Furthermore, the sample is subject to selection effects due to its reliance on publicly available court information, which skews toward the United States. Accordingly, observations regarding AML enforcement on mixers are heavily informed by U.S. policy and may not scale to other countries. Still, the dataset is the most comprehensive collection of publicly documented mixer money laundering cases assembled to date, from which we can derive preliminary patterns that can serve as a proof-of-concept for future research.

## Results

The following observations are drawn from the dataset of CML cases (*n* = 32), documented in the Appendix. By jurisdiction, there are 30 U.S. cases, 1 Canadian case, and 1 Dutch case. Twenty-two cases resulted in guilty verdicts or pleas, 7 are still ongoing at the time of this study, and 3 were dropped.

### Mixer Activity

The criminal activity of each recorded mixer is documented in Table 1, in terms of the number of cases involved and the total amount laundered. Monetary figures represent mixer-level, service-lifetime transaction volumes drawn from prosecution and related legal documents rather than amounts laundered in individual cases.

**Table 1.** Prevalence of Different Cryptocurrency Mixers Involved in Documented CML Cases.

Mixer name	Number of cases involved	Total transferred (USD)	Total from illegal sources (USD)
Tornado Cash	3	7,000,000,000	2,200,000,000
Helix	2	311,000,000	121,500,000
Bitcoin Fog	2	400,000,000	400,000,000
Samourai Wallet	2	2,000,000,000	100,000,000
Wasabi Wallet	2	36,000,000	36,000,000
Blender.io	2	500,000,000	20,500,000
ChipMixer	1	3,000,000,000	2,000,000,000
Cryptonator	1	1,400,000,000	235,000,000
BestMixer	1	275,000,000	—
Bitmixer.io	1	—	—
Sinbad.io	1	655,400,000	655,400,000
Silk Road tumbler	1	1,280,000,000	1,280,000,000
Silk Road 2.0 tumbler	1	8,000,000	8,000,000
AlphaBay tumbler	1	450,000,000	450,000,000
Unknown	13	—	—
Total	34	17,315,400,000	7,506,400,000

*Note.* Two cases both involved two different mixers. Figures associated with ongoing or dismissed proceedings are alleged and have not been judicially established.

The data indicate a wide distribution of mixers involved in documented CML cases, with large transfer amounts concentrated among a few services. Not much else can be drawn from the frequency of mixers in cases, given the limited sample size. Tornado Cash was the mixer most frequently implicated, transferring around $7 billion worth of cryptocurrency in this sample, with $2.2 billion from illegal sources. Other notably active mixers include Bitcoin Fog ($400 million confirmed transferred, all from illegal sources), Samourai Wallet ($2 billion transferred, at least $100 million from illegal sources), Blender.io ($500 million transferred, at least $20.5 million from illegal sources), ChipMixer ($3 billion transferred, at least $2 billion from illegal sources), Cryptonator ($1.4 billion transferred, at least $235 million from illegal sources), Sinbad.io ($655.4 million transferred, all from illegal sources), the Silk Road tumbler ($1.28 billion transferred, all from illegal sources), and the AlphaBay tumbler ($450 million transferred, all from illegal sources). Relatively small transfer amounts were documented for the Silk Road 2.0 tumbler and Wasabi Wallet ($8 million and $36 million, respectively), with all their documented funds involving illicit origins. However, Wasabi Wallet’s low transfer volume is likely due to the study’s methodological constraints, to which we will return in the Discussion. Despite Bitmixer.io having been one of the largest Bitcoin mixers while it was active, there is no reliable, publicly available information on its transfer volume. The identity of the mixer(s) involved in 13 cases was also unavailable.

### Custodial and Non-Custodial Mixers

Most mixers are custodial; only Tornado Cash, Wasabi Wallet, and Samourai Wallet can be classified as non-custodial, allowing users to retain control of their assets. Wasabi Wallet used CoinJoin, rendering it a centralized mixing service with a decentralized protocol (Wu et al., 2021). Samourai Wallet ran similarly. As such, Wasabi Wallet and Samourai Wallet are subcategorized as coordinator-based, non-custodial mixers. Tornado Cash is fully decentralized, as the mixer itself has no controlling operator. Criminal activity of custodial mixers relative to non-custodial mixers, in terms of total amount laundered, is recorded in Table 2.

**Table 2.** Total Amount Laundered With Custodial Versus Non-Custodial Cryptocurrency Mixers.

Type	Total transferred (USD)	Total from illegal sources (USD)
Centralized custodial	8,279,400,000	5,170,400,000
Non-custodial subtotal	9,036,000,000	2,336,000,000
Coordinator-based	2,036,000,000	136,000,000
Fully decentralized	7,000,000,000	2,200,000,000
Total	17,315,400,000	7,506,400,000

*Note.* Cases involving unknown mixers (*n* = 13) were not included. Figures associated with ongoing or dismissed proceedings are alleged and have not been judicially established.

Although only 3 out of 14 mixers are non-custodial, they account for a larger share of total cryptocurrency laundered. Of the documented $17.3 billion that was transferred, non-custodial services were responsible for $9.04 billion (52%), while centralized mixers transferred $8.28 billion (48%). Coordinator-based, non-custodial services accounted for $2.04 billion (12%), while the fully decentralized category, represented by Tornado Cash, accounted for $7 billion (40%). However, centralized mixers were responsible for an even larger proportion of the total $7.5 billion transferred from illicit sources, laundering $5.17 billion (69%) compared to $2.34 billion (31%) for non-custodial services ($2.2 billion was fully decentralized and $136 million was coordinator-based, non-custodial).

### Timeline of Mixer Activity

Longitudinal analysis of activity for mixers is displayed in Figure 1.

**Figure 1.**
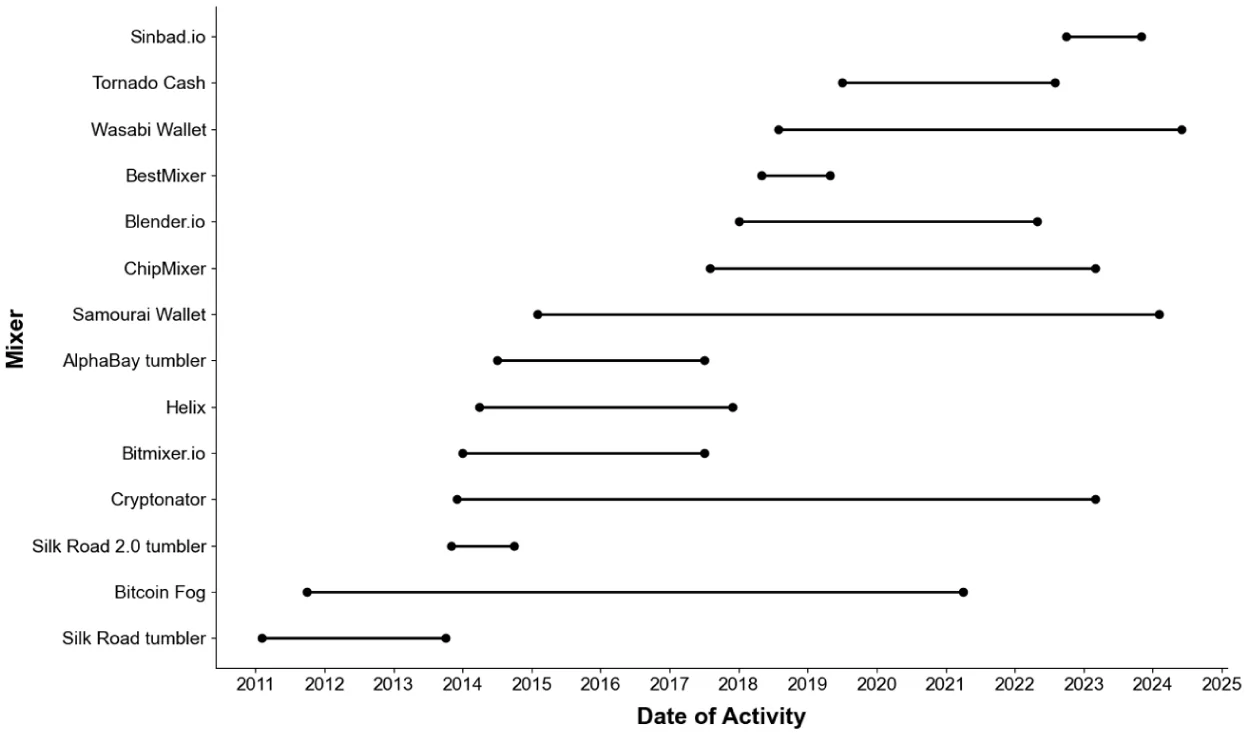
Timeline of activity for different cryptocurrency mixers. *Note.* Wasabi Wallet’s CoinJoin mixing services were deactivated, but the wallet is still currently active. While Tornado Cash was sanctioned and its centralized web-based interface taken offline, it is technically still active due to its decentralized infrastructure.

Activity was sparse during the early 2010s, with only two active mixers throughout 2011: the Silk Road tumbler and Bitcoin Fog. Mid-decade, the number of active mixers spiked with the development of Cryptonator, Bitmixer.io, and Helix, peaking at eight active mixers in 2018. Activity leveled off as major mixers were shut down and fewer new centralized mixers emerged, eventually leveling off at only two active mixers in 2024. Overall, mixer activity was most prevalent from 2014 to 2022. We will situate these trends in their historical and regulatory contexts in the Discussion.

### Mixer Countries of Origin

Table 3 records the country of origin for mixers involved in the documented CML cases. Country of origin was determined as a function of the jurisdiction where the company was incorporated, where the mixer’s online servers were located, or the mixer’s primary location of operation. Accordingly, Table 3 should be interpreted as mapping documented geographic affiliations rather than identifying a single, absolute country of origin, and any inferences should be understood in that context.

**Table 3.** Locations of Different Cryptocurrency Mixers.

Country	Number of mixers	Affiliated mixers
Germany	2	ChipMixer, Cryptonator
The Netherlands	2	BestMixer, Sinbad.io
The United States	1	Helix
The United Kingdom	1	Samourai Wallet
Seychelles	1	Wasabi Wallet
Russia	1	Blender.io
Luxembourg	1	BestMixer
Finland	1	Sinbad.io
Iceland	1	Silk Road tumbler
Lithuania	1	Silk Road 2.0 tumbler
Thailand	1	AlphaBay tumbler
Unknown/not applicable	3	Tornado Cash, Bitcoin Fog, Bitmixer.io

*Note.* BestMixer and Sinbad.io both had servers located in two different countries.

Germany and the Netherlands emerge as the most frequent countries of origin, each hosting two mixers: Germany with ChipMixer and Cryptonator, and the Netherlands with BestMixer and Sinbad.io. Countries of origin for Bitcoin Fog and Bitmixer.io are uncertain. Tornado Cash does not have a location, given that it is not a centralized entity. The rest of the mixers are evenly distributed among the United States, the United Kingdom, the Seychelles, Russia, Luxembourg, Finland, Iceland, Lithuania, and Thailand. The presence of Germany, Russia, the United States, and the United Kingdom is unsurprising. However, the presence of Luxembourg, Iceland, and the Seychelles is notable, as they are relatively small marketplaces. Nevertheless, the relatively small sample size thwarts generalizable inferences.

### Cryptocurrencies Affiliated With Mixer Activity

The types of virtual currencies involved in CML schemes may offer insight into users’ preferences and technical affordances that are exploited by illicit actors. The most commonly used cryptocurrencies for CML are visualized in Figure 2.

**Figure 2.**
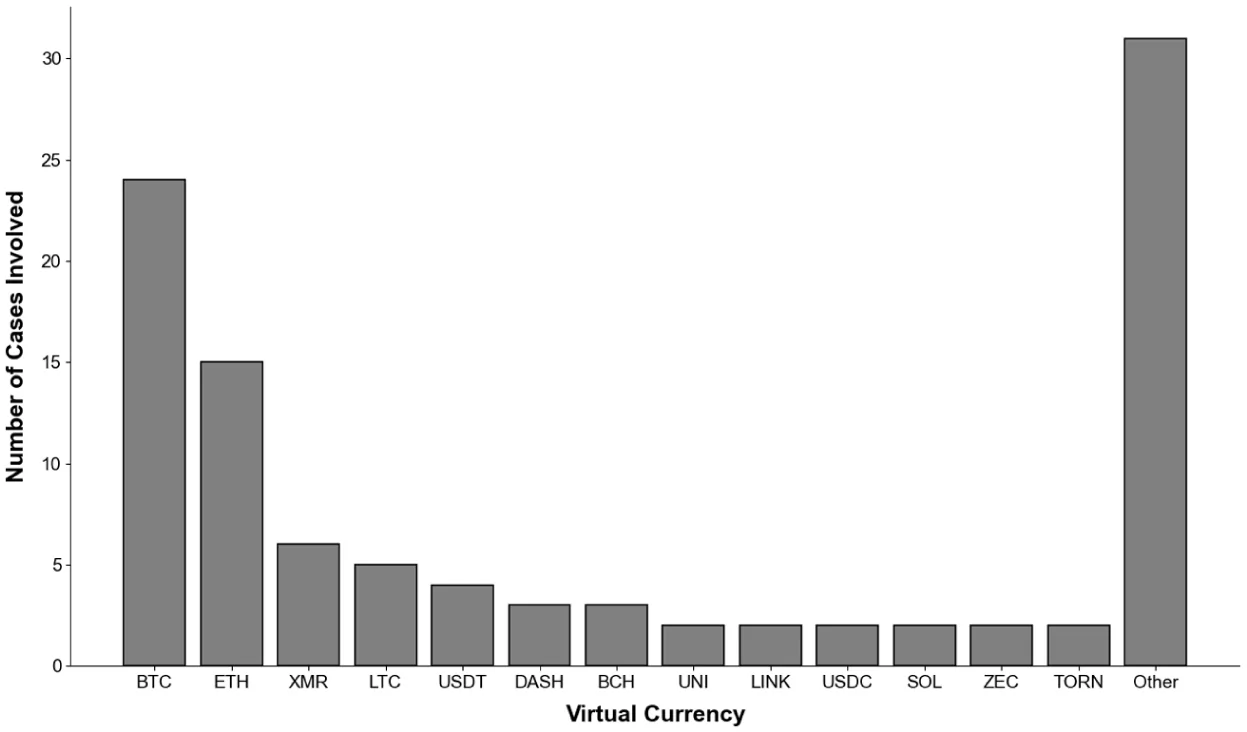
Prevalence of different cryptocurrencies involved in documented CML mixer cases. *Note.* From left to right: Bitcoin, Ether, Monero, Litecoin, Tether, Dash, Bitcoin Cash, Uniswap, Chainlink, USDC, Solana, Zcash, Tornado Cash. Virtual currencies that appear only once are included in “Other” (*n* = 31).

Bitcoin is by far the most common, appearing in 24 cases, which is consistent with its long-standing status as the most widely used and accepted cryptocurrency. Appearing in 15 cases, Ether follows a distant second, though still well ahead of other cryptocurrencies. Monero, a privacy coin designed to obscure transactions, was involved in six cases, followed by Litecoin with 5 cases, Tether with 4 cases, and Dash and Bitcoin Cash both with three cases. Uniswap, Chainlink, USDC, Solana, Zcash, and Tornado Cash token were all involved in 2 cases. Many unique cryptocurrencies (31) appeared only once across all cases, reflecting diversity in virtual assets used for laundering.

## Discussion

Results are likely underestimates: total amounts laundered by some mixers may not represent the true total amount due to a lack of open-source data. Wasabi Wallet is a prominent example since it undoubtedly transferred more than $36 million in cryptocurrency both from illegal sources and in total. Accordingly, amounts should be treated as minimums. Overrepresentation of U.S. cases (94%) could be a function of more readily accessible U.S. legal databases, its dominant global financial position, its prominent role in global cryptocurrency activity (Chainalysis, 2024b), and/or rigorous AML enforcement.

In addition, the data suggest that a small subset of mixers is responsible for laundering illegal funds, with just five mixers linked to over $6 billion transferred in illicit cryptocurrency. This could reflect a preference among cryptocurrency launderers for select reliable mixers. Enforcement may only need to target a few mixers to have an outsized impact. Tornado Cash alone was involved in three major court cases, transferring a total of over $7 billion in cryptocurrency, with $2.2 billion from illegal sources. This aligns with global observations that Tornado Cash was a prominent mixer of choice for concealing illicit transactions, particularly for state-based hackers such as Lazarus Group (Chainalysis, 2024a). Even after enforcement action, Tornado Cash remains a CML liability, with over 25% of its funds linked to illicit activity (Chainalysis, 2025). ChipMixer and the Silk Road tumbler also laundered significant amounts from illicit sources ($2 billion and $1.28 billion, respectively). Since the Silk Road tumbler operated for the Silk Road darknet market, all its transferred cryptocurrency came from illegal sources (akin to the Silk Road 2.0 tumbler and AlphaBay tumbler), which allowed these services to be shut down without affecting law-abiding users. Darknet markets are commercial websites that are commonly used to trade in narcotics, stolen assets, and illicit weapons. They operate on the dark web, which is accessible only through privacy-enhancing software, such as the Tor Browser. As will be seen, darknet markets recur as customers for mixers.

The custodial–non-custodial dynamic among mixers is worthy of note: although only three out of 14 mixers were non-custodial (Tornado Cash, Wasabi Wallet, and Samourai Wallet), they accounted for the majority of transferred cryptocurrency (52%). One might expect a preference for non-custodial mixers due to their resilience to being shut down, as seen with Tornado Cash’s recent resurgence following its sanctioning and web-based interface being taken down (Chainalysis, 2025; See, 2023). Indeed, Tornado Cash’s massive transfer volume ($7 billion) forms the bulk of the cryptocurrency transferred by non-custodial mixers. However, for confirmed illicit funds, centralized mixers out-launder non-custodial mixers 69% to 31%. This disparity suggests a criminal preference for centralized services, possibly because they are more traditional and easier to use (Mariani & Homoliak, 2025). However, it is puzzling that these reasons should outweigh the superior security benefits of non-custodial mixers. Alternatively, this difference may be a function of the ease with which authorities can trace and identify transactions from illegal sources with custodial mixers relative to non-custodial ones (See, 2023). Nevertheless, we can conclude that both custodial and non-custodial mixers have significant criminogenic attributes. Given that only three mixers were non-custodial, their substantial share of illicit funds transferred indicates that decentralized protocols play an outsized role in CML.

Perhaps most relevant to regulatory and enforcement actions is a mixer’s timeline of activity. The sparse prevalence of mixers in the early 2010s corresponded with the early stages of cryptocurrency; Bitcoin was the first cryptocurrency created in 2009 (Song et al., 2022). The subsequent rise in activity was likely due to growing demand for cryptocurrency: a surge in mixers mid-decade coincides with Bitcoin’s growth in popularity in 2014, and the peak of eight active mixers in 2018 mirrors both Bitcoin’s boom to $3.2 trillion in transactions and a spike in Ethereum that year (Song et al., 2022). After 2018, there was a large drop-off as U.S. AML enforcement intensified and mixers were taken down by law enforcement, including BestMixer, Bitcoin Fog, and Blender.io. Increased enforcement has also had a deterrent effect, with Bitmixer.io and Wasabi Wallet voluntarily closing their mixing services, citing concerns over the legality of mixers (Canellis, 2025; U.S. Secret Service, 2025). However, the vast majority of mixing services had to be forcibly taken down by law enforcement, which reflects their cryptoanarchist philosophy (Chohan, 2017b). This belligerent attitude is exemplified by Sinbad.io, which lasted only a year after opening as a replacement when Blender.io was shut down—a recurring pattern with mixers. Although mixers remain a significant ongoing area of CML concern, handling $504.3 million in illicit cryptocurrency in 2023, the golden age of cryptocurrency mixers seems to be over in part due to law enforcement efforts and cybercriminals pivoting to alternate methods, such as cross-chain laundering and gambling platforms (Chainalysis, 2024a).

Data on the mixers’ jurisdictions of operation also yield regulatory implications, although the small sample size precludes broad conclusions. Germany and the Netherlands both hosted two mixers each: the former hosted ChipMixer and Cryptonator, and the latter hosted BestMixer and Sinbad.io. Although both countries have strong compliance with the FATF AML recommendations and active enforcement of cryptocurrency regulations (FATF, 2024a, 2024b), they also have large financial marketplaces, technological infrastructures, and user bases, which may explain their affinity with mixers. Russia, the United Kingdom, and the United States have large financial marketplaces and cryptocurrency user bases (Chainalysis, 2024b). Finland’s relatively small population and historically conservative regulatory approach make its affiliation with Sinbad.io noteworthy. This finding confirms other research identifying Finland as a CML outlier. For example, in 2019, cryptocurrency exchanges in Finland received the highest percentage of funds from illicit sources at 12.1%, while the global average was only 0.17% (Hou, 2022). However, this may be a function of Finland’s historically influential role in shaping the cryptocurrency ecosystem, helping develop Bitcoin and founding the Aave open-source protocol (Viitaharju, 2024). Noteworthy are smaller countries such as Luxembourg, Iceland, and the Seychelles, affiliated with BestMixer, the Silk Road tumbler, and Wasabi Wallet, respectively. Given that these countries have historically had weak cryptocurrency regulatory oversight, this observation suggests regulatory arbitrage by operators to exploit “safe haven” jurisdictions, which underscores the importance of global regulatory compliance and cooperation.

Bitcoin dominates as the most frequently used cryptocurrency in mixer CML cases (24), distantly followed by Ether (15), Monero (6), and Litecoin (5). This observation aligns with Leuprecht et al.’s (2023) analysis of 12 general CML cases, which found that Bitcoin was by far the most used cryptocurrency, again followed by Ether and Litecoin. Bitcoin remains the most widely accepted and liquid cryptocurrency, and criminals often need to use Bitcoin to enter or exit the crypto economy. Many darknet markets are priced in Bitcoin, with markets receiving over $2 billion in Bitcoin in 2024 (Chainalysis, 2025), and many exchanges traditionally only dealt in Bitcoin, forcing criminals either to start with Bitcoin or convert into Bitcoin to cash out. Ether’s prevalence reflects its growing role as a prominent cryptocurrency for CML, especially in recent scams related to NFTs, which are cryptographic tokens primarily created on the Ethereum network (Ozga & Leuprecht, 2026). Our results point to a heterogeneous cryptocurrency ecosystem, with 31 unique altcoins appearing only once across all cases. Altcoins are any virtual currencies that are not Bitcoin. This suggests that launderers are willing to use whatever assets are available or least monitored in a given context, though they are usually ancillary to the main Bitcoin/Ether flows. Privacy-focused altcoins are also present but not yet dominant, such as Monero (6), Dash (3), and Zcash (2). These coins offer built-in anonymity, so one might expect greater usage, but their adoption is likely limited by the fact that relatively few exchanges and merchants accept them. Criminals may use privacy coins for an intermediate layer of obfuscation. Ultimately, they often must convert back to Bitcoin or fiat at some point, which reintroduces exposure to authorities and investigators. The following section further illustrates these patterns.

### Three Representative Case Studies

To synthesize the CML mixer cases, we classified each case into one of three categories based on the defendant’s relation to the mixer:

1. *Overseer* cases involve individuals or groups that develop, sustain, and/or run the mixer, allowing others to use their service to launder cryptocurrency.
2. *Personal user* cases involve individuals or groups that use mixers as customers to help launder their cryptocurrency gains; they do not own or control the mixer.
3. *Enabler* cases involve individuals or groups that run cryptocurrency exchanges without proper due diligence or AML safeguards, thereby facilitating CML transactions involving mixers.

Our dataset documents 11 overseer cases, 18 personal user cases, and 3 enabler cases. A representative case study is illustrated for each case type below.

The overseer case involves Larry Dean Harmon, the operator of the Helix mixing service, which reportedly laundered over $311 million in cryptocurrency from 2014 to 2017. He was charged by the U.S. Department of Justice with money laundering conspiracy and operating an unlicensed money transmitting business—common charges for overseer cases—and later pleaded guilty to the first charge. According to court documents, Harmon advertised Helix to darknet market customers to conceal transactions from law enforcement. He reportedly later partnered with darknet market AlphaBay and aided customers in obfuscating cryptocurrency transactions involving guns, drugs, and other illegal goods and services, earning a profit by taking a percentage of transactions as a fee. Other darknet markets that used Helix include Agora, Nucleus Marketplace, and Dream Market. Helix did not register under the *Bank Secrecy Act*, report suspicious activity, or implement any AML procedures, highlighting blatant disregard for AML regulations shared by many mixer operators.

The personal user case involves Ilya Lichtenstein and Heather Morgan. Allegedly, they committed wire fraud, computer fraud and abuse, and money laundering conspiracy; both pleaded guilty to the latter charge. According to the U.S. criminal investigation, in August 2016, Lichtenstein breached the security of Bitfinex, a major cryptocurrency exchange, and stole $71 million worth of Bitcoin. Enlisting Morgan, he subsequently hid the stolen funds by transferring them across cryptocurrency wallets, darknet market accounts at AlphaBay, and accounts at 10 different cryptocurrency exchanges. Lichtenstein testified to using Bitcoin Fog and other mixers to obfuscate their transaction trails. To enhance the CML process, they also employed fictitious identities to create accounts at exchanges, transferred stolen funds in small amounts over thousands of transactions, and converted Bitcoin into other forms of cryptocurrency, including privacy coins. Five years on, they were ultimately caught after investigators used blockchain analysis tools to trace stolen funds to an account connected to Lichtenstein’s real identity. In addition to hacks and darknet market transactions, another common source of funds for personal user cases is investment scams, whereby fraudsters promote seemingly legitimate cryptocurrency projects, abruptly abandon them after extracting investor liquidity in a “rug pull,” and launder the stolen assets through mixers to obscure their origin (see “E. Nguyen, A. Llacuna” and “A. Michel” cases in Appendix).

The third case type, enabler cases, involves Alexander Vinnik, who was charged with operating an unlicensed money-transmitting business, money laundering, money laundering conspiracy, and engaging in unlawful money transactions; he later pled guilty to money laundering conspiracy. Vinnik operated BTC-e, a large cryptocurrency exchange that served millions of users and processed over $9 billion in transactions. According to U.S. prosecutors, BTC-e did not register with FinCEN nor apply any AML/KYC policies, enabling cybercriminals to launder illicit proceeds from hacking and ransomware attacks, identity theft schemes, and narcotics distribution rings. BTC-e also reportedly received millions of dollars in cryptocurrency from AlphaBay and Hydra darknet markets, non-compliant exchanges, and unnamed Bitcoin mixing services. The most frequently exchanged cryptocurrencies on the platform were Bitcoin and Litecoin, while U.S. dollars and Russian rubles were the most common fiat currencies. Despite BTC-e publicly advertising that they did not accept U.S. customers or wire transfers, they knowingly accepted transactions from U.S. banks and citizens. This represents a common pattern among both mixer and exchange operators: they publicly exit the U.S. market to avoid complying with regulators while still covertly marketing to U.S. customers, leaving them exposed to law enforcement action. Vinnik was ultimately released in February 2025 by the United States in a prisoner exchange with Russia (Betts, 2025).

### Implications

Findings raise significant regulatory and enforcement challenges, particularly given the resilience of non-custodial mixers combined with their high laundering volumes. Unlike custodial services that have identifiable operators, decentralized mixer protocols lack a central nexus and are exceedingly difficult to shut down, as exemplified by the recent resurgence of Tornado Cash (Brownworth et al., 2024; Chainalysis, 2025). Moreover, illicit mixers operate through an incessant “whack-a-mole” pattern: when one mixer is shut down, others rapidly take its place, as seen with Blender.io and Sinbad.io. As a result, while illicit use of mixers has been on the wane (Chainalysis, 2024a), sustained impact remains elusive in the face of mixers’ built-in resilience and the adaptive strategies of cybercriminals.

These findings also highlight the value of examining CML through empirical court data rather than technical transaction analyses alone. They demonstrate that within a broader ecosystem of diverse virtual assets, CML is concentrated among a small number of mixing services, while decentralized infrastructures are resilient against traditional enforcement. These patterns reinforce the extent to which CML differs from conventional money laundering. This necessitates enforcement strategies to account for decentralized financial technologies.

Equally important are implications stemming from the typologies of actors associated with CML mixing. Our research categorizes cases into three agent-centered groups: overseers who operate mixing services, personal users who use mixers to launder their own illicit proceeds, and enablers who operate exchanges and facilitate mixer laundering through a lack of AML/KYC protections. This broad spectrum of roles of actors illustrates that CML is a multifaceted ecosystem, and thus enforcement should not target illicit mixer services on their own. Indeed, despite many countries requiring AML regulations for cryptocurrency exchanges, they remain essential players at the beginning and end of the CML process (van Wegberg et al., 2018; Zola et al., 2025). As darknet markets and hackers continue to play a prominent role in sourcing CML, enforcement policy should shift from primarily responsive action to preventive measures, such as proactively ensuring AML/KYC policies are in place before a mixer or exchange has already been used to launder millions in cryptocurrency.

The article’s results also carry broader regulatory implications concerning international coordination and the technological scope of AML enforcement. The global dispersion of mixer operators illustrates how inconsistencies in national regulatory regimes encourage regulatory arbitrage: several major mixers are situated in jurisdictions with weak cryptocurrency oversight (e.g., the Seychelles, Luxembourg, and Iceland). Notably, 27% of countries have not implemented requirements for VASPs to obtain sender/recipient information for cryptocurrency transactions, and 24% have not conducted any VASP risk assessments (FATF, 2025). Given that VASPs function as the primary off-ramp in CML schemes, these regulatory gaps call for improved international AML coordination. In addition, the presence of altcoins in CML schemes exposes a technological asymmetry in enforcement capabilities, as law enforcement tools have historically been trained to trace Bitcoin; tracking privacy coins incurs greater transaction costs (Leuprecht et al., 2023). Finally, cryptocurrency policies must account for the cryptoanarchist philosophy that informs mixers (Chohan, 2017b). Mixer-takedown patterns suggest that few mixers will voluntarily shut down out of pragmatic or monetary concerns, calling into question the efficacy of approaches primarily aimed at creating a deterrent effect.

## Conclusion

This article examined the role of cryptocurrency mixers in money laundering through a comparative analysis of enforcement actions. It offers an empirically grounded perspective on a phenomenon that has hitherto been discussed in largely technical or speculative terms. Findings demonstrate that mixer-enabled CML is neither diffuse nor random. Instead, it is concentrated among a small number of services, involves recurring actor roles, and persistently relies on established cryptocurrencies, such as Bitcoin and Ether. While non-custodial mixers accounted for a substantial share of the overall volume of transactions, custodial mixers were responsible for the majority of funds traced directly to illicit sources. That underscores the continued salience of custodial services within criminal ecosystems.

Results highlight the structural limits of reactive enforcement strategies that prioritize takedowns and prosecutions after substantial harm has already been done. The rapid substitution of mixing services following enforcement actions, combined with jurisdictional fragmentation and uneven regulatory capacity, reflects an adaptive offender environment that consistently outpaces formal controls. The identification of overseers, personal users, and enablers further posits mixer-based laundering as a networked process that spans service providers, offenders, and gatekeepers as opposed to a discrete or easily isolated activity. Effective responses to decentralized financial technologies necessitate moving beyond traditional assumptions about control, jurisdiction, and deterrence.

Cryptocurrency mixers constitute a structurally embedded and adaptive component of contemporary financial crime rather than a peripheral or transient phenomenon. While enforcement actions have demonstrated some capacity to disrupt specific services, their overall effectiveness remains constrained by the resilience of decentralized protocols, the rapid substitution of platforms, and persistent jurisdictional gaps. These dynamics highlight the limits of approaches that are centered primarily on reactive punitive measures and prosecutions. Instead, addressing mixer-facilitated laundering requires a recalibration of criminal justice strategies, which will involve proactive regulation, strengthened oversight of VASPs, and deeper international coordination. Cryptocurrency mixers illustrate how emerging financial technologies reconfigure opportunity structures for crime, calling for renewed criminological attention to the evolving relationship between innovation and illicit enterprise.

## Appendix

**Appendix:** Documented CML Court Cases Involving Cryptocurrency Mixers.

Defendant(s)	Jurisdiction	Outcome	Year charged	Mixer(s) involved	Case type
L. D. Harmon	USA	Guilty	2019	Helix	Overseer
R. Storm, R. Semenov	USA	Ongoing	2023	Tornado Cash	Overseer
R. Sterlingov	USA	Guilty	2021	Bitcoin Fog	Overseer
K. Rodriguez, W. L. Hill	USA	Guilty	2024	Samourai Wallet	Overseer
M. Q. Nguyen	USA	Ongoing	2023	ChipMixer	Overseer
A. Pertsev	Netherlands	Guilty	2022	Tornado Cash	Overseer
C. Castelluzzo, L. Atwell	USA	Guilty	2013	Unknown	Personal user
E. Nguyen, A. Llacuna	USA	Guilty	2022	Tornado Cash	Personal user
I. Lichtenstein, H. Morgan	USA	Guilty	2022	Bitcoin Fog	Personal user
J. J. O’Connor	USA	Guilty	2021	Unknown	Personal user
R. J. Schueler	USA	Dropped	2023	Unknown	Personal user
S. Ahmed	USA	Guilty	2023	Samourai Wallet	Personal user
D. G. Saffron, V. A. Mazzotta Jr.	USA	Ongoing	2023	Unknown	Personal user
BTC-e, A. Vinnik	USA	Guilty	2017	Unknown	Enabler
R. W. Ulbricht	USA	Guilty	2014	Silk Road tumbler	Overseer
R. Boss	USA	Dropped	2024	Cryptonator, Blender.io	Overseer
P. Engstrom, V. Cuomo, A. Elliott, J. Krieger	USA	Guilty	2021	Unknown	Personal user
A. Michel	USA	Guilty	2023	Unknown	Personal user
J. Zhong	USA	Guilty	2022	Unknown	Personal user
S. Vachon-Desjardins	Canada	Guilty	2021	Unknown	Personal user
G. He, Z. Wang	USA	Ongoing	2022	Wasabi Wallet	Personal user
V. Kvashuk	USA	Guilty	2019	Unknown	Personal user
C. Zhao	USA	Guilty	2023	BestMixer	Enabler
B. Benthall	USA	Guilty	2014	Silk Road 2.0 tumbler	Overseer
C. A. Boden, L. B. Vogt, D. R. Dejager	USA	Guilty	2021	Unknown	Personal user
H. Adams, D. Hosner	USA	Guilty	2022	Unknown	Personal user
K. Sirotkin, K. Stephens, S. Deaver, A. Jones, S. Sirotkin	USA	Guilty	2019	Wasabi Wallet	Personal user
A. Cazes	USA	Dropped	2017	AlphaBay tumbler	Overseer
D. Kaye	USA	Ongoing	2021	Bitmixer.io	Personal user
A. Medjedovic	USA	Ongoing	2024	Unknown	Personal user
HDR Global Trading Limited	USA	Guilty	2020	Helix	Enabler
R. V. Ostapenko, A. E. Oleynik, A. V. Tarasov	USA	Ongoing	2025	Sinbad.io, Blender.io	Overseer
